# Physiological and Immunological Changes Associated with Oral Microbiota When Using a Thermoplastic Retainer

**DOI:** 10.3390/molecules26071948

**Published:** 2021-03-30

**Authors:** Wurood kh. Al-lehaibi, Khulood A. Al-makhzomi, Hani Sh. Mohammed, Hamid Hammad Enezei, Mohammad Khursheed Alam

**Affiliations:** 1 Department of Pedodontic, Orthodontic and Preventive Dentistry, Dentistry Department, Dijlah University College, Baghdad 10011, Iraq; 2 Department of Pedodontic, Orthodontic and Preventive Dentistry, College of Dentistry, Uruk University, Baghdad 10069, Iraq; dr_kholood_almakhzomi@yahoo.com; 3 Department of Oral Diagnosis, Al Ramadi Specialized Dental Center, Ministry of Health, Ramadi 31001, Iraq; Hanishareef76@gmail.com; 4 Department of Oral & Maxillofacial Surgery, College of Dentistry, University of Anbar, Ramadi 31001, Iraq; den.hamed.hamad@uoanbar.edu.iq; 5 Orthodontic Division, Preventive Dentistry Department, College of Dentistry, Jouf University, Sakaka 72345, Saudi Arabia

**Keywords:** thermoplastic retainer, salivary pH, IgA, oral microbiota, adhesion, chlorhexdine, *S. mutans*, *Lactobacillus* spp.

## Abstract

*Background*: The study examined the oral microbiota, physiological and immunological changes in patients using thermoplastic retainers during three months of use. *Methods*: The study included several steps. Firstly, 10 swabs were collected from the buccal and palatal surfaces of the teeth of the patients, approximately 2 mL of saliva was collected from the same patients and 2 mL of saliva was collected from 10 healthy people to measure the pH and secretory IgA level. This was followed by the isolation and identfication of the bacterial isolates in the patient samples. Then, isolate susceptibility toward chlorhexidine (CHX) and their adhesion ability to thermoplastic retainer surfaces was measured. In addition to that the study estimated the numbers of *Lactobacillus* and *Streptooccus mutans* colonies during three months and finally, a comparsion of pH acidity and IgA level between the patients and healthy people was performed. The results showed the predominant bacteria during the three months were *Lactobacillus* spp. and *Streptococcus* spp. followed by different rates of other bacteria. *Raoultella ornithinolytica* showed more resistance to CHX while *Lactobacillus* spp. showed more sensitivity. *Streptococcus mutans* colony levels were higher than *Lactobacillus* spp. colonies during the three months, also *S*. *mutans* had the highest value in adherence to retainer thermoplastic. Finally, pH acidity showed a highly significant difference (*p* ≤ 0.05) in the third month, like IgA levels (*p* ≤ 0.05). *Conclusions*: According to the results obtained from the current study, the researchers noted that the thermoplastic retainers helped change the oral cavity environment.

## 1. Introduction

The term oral microbiome refers to the collective genome of oral cavity microorganisms. It is the second-largest microbial species in humans after the intestinal microbiome, and they have an amazing variety of predicted protein functions compared with other body sites. The human microbiome is made up of a main and a variable microbiome. The basic microbiome is common to all people, while the variable microbiome depends on the host lifestyle and physiological variations. The oral cavity has two kinds of surface which bacteria can colonize, the strong and the soft teeth, as well as the oral mucosa [1]. Over 700 bacterial species co-inhabit a normal healthy human mouth. Broadly these microorganisms belong to the genera *Lactobacillus*, *Streptococcus*, *Eubacteria*, *Fusobacterium*, *Capnocytophaga*, *Eubacteria*, *Staphylococcus*, *Eikenella*, *Porphyromona*, *Leptotrichia*, *Prevotella*, *Peptostreptococcus*, *Treponema* and *Actinomyces* [2]. Oral microbiomes can display significant and rapid changes in spatial and time composition and activity and their growth is host-dynamic. The multiple and non-balanced dynamics are the product of numerous factors such as the temporal host and diet frequency, the response to pH changes, bacterial interactions, gene mutations, and horizontal gene transfers that extend new properties to strains over a longer time [3]. Microbiomes in the oral cavity may changed due to changes in the oral cavity environment, especially in patients who wear removable orthodontic appliances, regardless of the type of removable orthodontic appliance. The most commonly used retainers are three types: Hawley retainers made of metal and acrylic, Essix retainers, made from a polypropylene or polyvinyl chloride sheet and permanent retainers, which are metal wires fixed to the lingual surfaces of the teeth. These orthodontic devices are a popular and successful method for malocclusion treatment but can be linked to secondary effects including microbiome shifts and subsequent infections [4]. It is known that when removable orthodontic appliances are inserted in the oral cavity, they begin to accumulate plaque, but is not known whether the accumulation of plaque depends on the material from which the device is made because most studies in the literature do not specify it. The microorganism load increment could be strictly related to the appliance surface roughness as well as the time spent in the oral cavity [5].

The complicated undercut of orthodontic devices makes it more difficult for keep teeth clean and causes plaque accumulation and restorations, the risk of white-spot injuries, dental caries, and periodontal complications that therefore have been suggested to result from changes in the oral microbiome [6]. Inserting orthodontic appliances into the oral cavity significantly changes the oral hygiene and increases the number of plaque retention areas. These changes in the oral environment are followed by an increase in bacterial concentration, changes in buffer capacity, pH acidity, and salivary flow rate [7], suggesting that orthodontic appliances help to create a favorable environment for the accumulation of microbiota and also food residues, which over time lead to caries or periodontal diseases, and finally provide a favorable environment for non-oral normal flora [8].

The appliances interfere with the oral hygiene and cover major parts of the tooth surface that consequently delivers less saliva, so the total microbial population will increase as well as an alteration in microflora composition has been reported in relation to orthodontic treatment [9]. In fact those removable orthodontic appliance (retainers) are constructed from different materials to which different bacteria will get adhered but generally two common bacteria, *S. mutans* and *L. acidophilus*, are the predominant bacteria in dental caries and deep dentin cavities with the orthodontic appliance. In any case it is clear that orthodontic appliances are responsible for a f othe oral hygiene [10,11,12,13,14,15,16] and promoting the growth and differentiation of microbial flora inside the oral cavity that might result in caries, white spot lesions, and gingival inflammation and this may affect the environmental physiology and immunology directly or indirectly as evidenced by increases in salivary pH acidity and or secretory IgA levels attributed to change in the microbiota [11]. The study aimed to investigate the microbial, immunological, and physiological changes in patients that used a thermoplastic retainer for three months of treatment.

## 2. Results

The results of the isolation and identification of the bacteria for three months showed *Lactobacillus* spp. and *Streptococcus* spp. represented the highest percentage of the oral microbiota during the three months, while the percentage of other isolates (Table 1) varied according to the formula below:

(1) $$\mathrm{Isolate}\;\mathrm{percentage}\;\%=\frac{\mathrm{Total}\;\mathrm{number}\;\mathrm{of}\;\mathrm{isolate}\;\mathrm{in}\;\mathrm{patients}}{\mathrm{Total}\;\mathrm{number}\;\mathrm{of}\;\mathrm{patients}}\times 100\%$$

**Figure 1:**
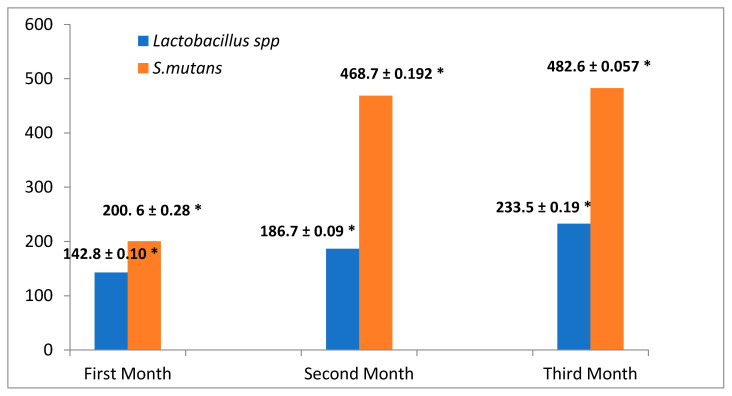
The comparison between the colony number between *S. mutans* and *Lactobacillus* spp. in patients with a thermoplastic retainer for three months (* highly significant).

The results revealed there were difference between the colony counts of *S.mutans* and *Lactobacillus* spp. whereby the number of *S. mutans* colonies was higher than that of *Lactobacillus* spp. colonies during the three months at 10^2^ dilution with significant differences (*p* ≤ 0.05). Thus in the first-month, the *S. mutans* colonies count was 200.6 ± 0.28, in the second month 468.7 ± 0.192 while in the third month it was 482.6 ± 0.057, whereas the number of *Lactobacillus* spp. colonies was also different: 142.8 ± 0.10 in in the first month, 186.7 ± 0.09 in the second month and finally in the third month 233.5 ± 0.19, also with significant differences (*p* ≤ 0.05) (Figure 1).

**Figure 2:**
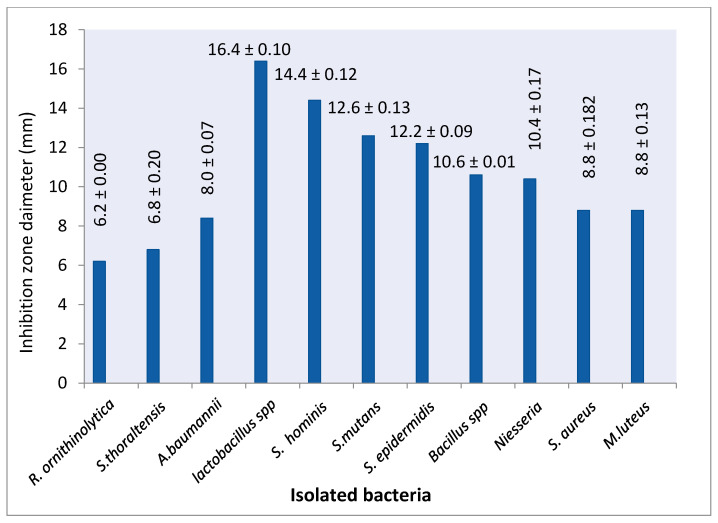
The inhibition zone diameter of CHX against isolated bacteria.

Moreover, the CHX sensitivity test revealed that the isolated bacteria showed various responses to CHX. *R. ornithinolytica* showed more resistance to CHX according to the diameter of the inhibition zone (6.2 ± 0.00 mm) followed by *S. thoraltensis* (6.8 ± 0.20) and *A. baumannii* (8.0 ± 0.07), while *Lactobacillus* spp. recorded the largest inhibition zone (16.4 ± 0.10) followed by *S. hominis* (14.4 ± 0.12), *S. mutans* (12.6 ± 0.13), *S. epidermidis* (12.2 ± 0.09), *Bacillus* spp. (10.6 ± 0.01), *Niesseria* spp. (10.4 ± 0.17), *S. aureus* (8.8 ± 0.182) and *M. luteus* (8.8 ± 0.13) (Figure 2).

**Figure 3:**
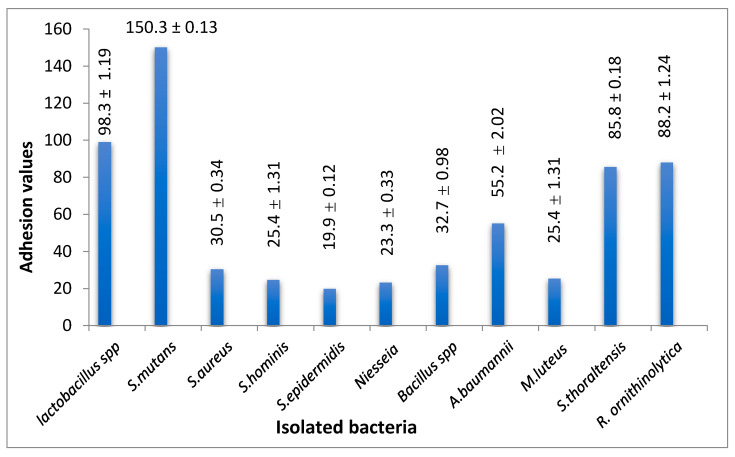
The adhesion ability of isolates on thermoplastic retainers during 1 h.

Furthermore, the adhesion ability of isolates to the thermoplastic retainer during 1 h showed that *S. mutans* had the highest value (150.3 ± 0.13) followed by *Lactobacillus* spp. (98.3 ± 1.19), *R. ornithinolytica* (88.2 ± 1.24), *S. thoraltensis* (85.8 ± 0.18), *A. baumannii* (55.2 ± 2.02), *Bacillus* spp. (32.7 ± 0.98), *S. aureus* (30.5 ± 0.34), *M. luteus* (25.4 ± 1.31), *S. hominis* (24.7 ± 0.02), *Niesseia* spp. (23.3 ± 0.33) and *S. epidermidis* (19.9 ± 0.12) with significant differences (*p* ≤ 0.05) between isolates (Figure 3).

Finally, the study investigated the physiological and immunological changes during three months, which included estimation of the salivary pH and IgA secretory level (Table 2). The results indicated there was a change in the pH salivary acidity, whereby the pH acidity in patients with thermoplastic retainers increased during the three-months in comparison with normal people with highly significant differences (*p* ≤ 0.05) in the third month (5.9± 0.26) and the second month (6.2 ± 0.312) in comparison to the first month (6.5 ± 0.112) and an increase in acidity compared to normal people (6.6 ± 0.02) but the difference was not significant (*p* ≤ 0.05). On the other had, the immunological results showed the concentration of secreted IgA in the third month recorded the highest value (13.8 ± 0.02) in comparison with the first month (13.8 ± 0.02) and the second month (13.4 ± 0.17) with highly significant differences but there was no significant differences between the second month and third month (*p* ≤ 0.05).

## 3. Discussion

The current study showed that both *Lactobacillus* spp. and *Streptococcus* spp. were the most predominant bacteria in the 10 patients during three months. Previous studies have described the main bacteria in the oral cavity is *Lactobacillus* spp. and *Streptococcus* spp., which are associated with dental caries. The location of the bacteria in saliva, tongue, carious lesions, dental plaque, etc. may play the main role in caries progression [16,17]. However, the oral microflora changed with time during the orthodontic treatment, with *S. mutans* and *Lactobacillus* spp. numbers increasing during the six months [18]. During the three months, *Staphylococcus* spp. was found in a higher ratio compared to the other isolates, Staphylococci are considered members of the transient oral microbiota and are seldom isolated from the oral cavity [19], the etiologic *Staphylococcus* spp. in the oral cavity were assumed to be acquired via a percutaneous route, associated with nosocomial infections, and those findings led us to assume that a portion of the causative *Staphylococcus* spp. in infective endocarditis originated in the oral cavity [20]. Moreover, the *Bacillus* spp. and *M. luteus* originally came into the oral cavity due to food consumption and hygiene habits [21,22]. On other hand the results recorded that non-oral pathogenic bacteria were isolated during the month like *R. ornithinolytica*. Other studies have reported the presence of non-oral pathogenic bacteria in the saliva of denture wearer patients at the same time as pathogenic bacteria, including Acinetobacter *Pseudomonas* spp. which are sources of contamination in dental laboratories [23,24]. Interestingly in genetic studies for the detection of bacteria in the oral cavity more non oral pathogenic isolates like *Acinetobacter* spp., *P. aeruginosa*, *Acinetobacter* spp., and *A. baumannii* were found, which are the major respiratory pathogens associated with nosocomial infections and transmission of these agents is attributed to person-to-person contagion, contaminated food, water, and hospital equipment [25,26]. Finally the presence of *S. thoraltensis* is noteworthy. This is an unusual species of streptococci that has recently been isolated from human samples taken from the nasal cavities or pharynx [27].

The alteration in the microbiota was attributed to the introduction of the thermoplastic retainers into the oral system that helped create a surface on which the bacterial species will be able to reproduce [28], insufficient saliva secretion and a resulting limitation of the antimicrobial effects of saliva [29]. On the other hand the colony counta of *Lactobacillus* spp. and *S. mutans* were increasing during the three months [30], also the number of *S. mutans* colonies was higher than that of *Lactobacillus* spp. colonies in agreement with the results reported by Teughels in [31]. *S. mutans* predominates in early plaque, but its concentration dropped in later weeks later [32]. The thermoplastic retainers might have a positive effect on *Lactobacillus* spp. and *S. mutans* colonization on dental surfaces. The decrease in *S. mutans* in the third month is attributed to the fact *S. mutans* are early colonizers of plaque because of the high oxygen concentrations they encounter, but ovetime as the plaque becomes more mature and more layers are added the oxygen levels will decrease and anaerobic flora such as Actinomyces species become dominant [33].

Moreover, the isolates showed various responses toward CHX. These different inhibition results are attributed to the different resistance mechanisms toward CHX. The antimicrobial effect of CHX is based on leakage of cytoplasmic materials due to damage to the bacteria cytoplasmic membrane [34]. The patients who attended the clinic during the sample collection period were treated with CHX, and the isolates showed resistance to CHX due to the long-term use of CHX, explained by genetic changes that favor the appearance of new clones of microorganisms with high resistance features [35].

Previous studies on the adhesion of bacteria on thermoplastic retainers have suggested the adhesion of microbes depends on the surface properties of the thermoplastic retainer and a high surface roughness leads to increased cell adhesion, while the surface energy, composition, surface hydrophobicity and zeta potential of the materials also influences the adhesion of cells [36]. The pH acidity was increased, which is attributed to the increase in the number of *Lactobacillus* which produce lactic acid to increase the acidity of the oral cavity as a defense mechanism against other bacteria [37]. In addition the secretory IgA level recorded the highest value in the second and third month [38]. The level of IgA is related with dental caries and associated with oral hygiene, and the reason is because an immune response is induced in the oral cavity by a high level of bacteria as well as presence non-oral opportunistic bacteria. One of the mechanisms of mucosal immunity is an increased secretion level of IgA in saliva [11]. Not only IgA was noticed to increase in these circumstances, as recently IL-6 was reported to increase, specially in children with poor oral hygiene performance indices, gingival inflammation and the presence of plaque [39].

## 4. Materials and Methods

### 4.1. Sample Collection

In this study, samples were collected from 10 orthodontic patients directly after they finished their treatment and received their vacuum-formed thermoplastic removable retainers, and for three months thereafter (one of the patients stopped attending the clinic in the second month). The sampling procedure included the collection of a bacterial swab from the buccal and palatal surfaces of the teeth for the isolation and identification of the bacteria, and *Lactobacillus* spp. and *S. mutans* colony counting, and approximately 2 mL of saliva was collected from the patients to measure the pH and secretory IgA. In addition to that, for comparison a sample of approximately 2 mL of saliva was collected from 10 healthy people (non-orthodontic patients).

### 4.2. Isolation and Identification of Bacteria

The swabs were inoculated in nutrient broth for 24 h and subcultured in different media, such as De Man, Rogosa and Sharpe agar, blood agar, bile esculin azide agar and mannitol salt agar. The isolates were identified by morphological characterization and biochemical tests such as carbohydrate fermentation tests, coagulase tests and oxidase tests [12] and confirmed by a Vitek 2 system (Olympus, Shinjuku-Ku, Japan).

### 4.3. Estimation of the Colony Counts of S. mutans and Lactobacillus *spp.*

*Lactobacillus* spp. colonies were counted by putting the swabs into 1 mL of trypticase soy broth and mixing gently, after which the suspensions were serially diluted to 1 × 10^2^. One mL from 1 × 10^2^ diluate was poured on plates containing Man, Rogosa, and Sharpe (MRS) agar and incubated for 24 h at 37 °C. For counting *S. mutans* colonies the same procedure was repeated but the diluted suspension [14] was poured on plates containing tryptone-yeast extra cysteine-sucrose-bacitracin agar from 1 to 3 days under microaerophilic conditions (air with 15% carbon dioxide) then the colony was counted using the viable colony count technique [13].

### 4.4. CHX Sensitivity

The isolates were prepared from nutrient broth (after 18- to 24-h) then adjusted to approximately 1.5 × 10^8^ CFU/mL, subcultured on Mullar Hinton agar contained in wells of equal size (6 mm in diameter) whereby each well was filled with 0.1 mL of CHX gluconate (2% *w*/*v*) [12] produced by Julphar Gulf Pharmaceutical Industries (Ras Al Khaimah, United Arab Emirates, 200 mL) obtained from a pharmacy in Baghdad, Iraq.

### 4.5. Determination of the Adhesion of Isolates on Thermoplastic Retainer Surfaces

The adhesion assay was used as described [15] with some modifications. The thermoplastic retainers was cut to segments (1 cm^2^) and placed in tubes with a 5-mL suspension of the tested bacteria; the mixture was incubated for 1 h at 37 °C and the thermoplastic retainer segments were washed three times with PBS, placed in 10 mL of fresh PBS and sonicated for 5 min at 40 kHz to dislodge the adherent cells. The sonicated PBS was serially diluted to 1 × 10^3^ and cultured on tryptic soy agar plates and the number of adherent bacteria determined by the viable colony count technique.

### 4.6. Measurement of Salivary pH

The salivary pH saliva was estimated immediately using a pH meter (Radiometer, Crawley, UK). The pH meter was calibrated using freshly prepared buffers of pH 7 and the electrode was kept dipped in double-distilled water when not in use. After analyzing the pH, the electrode tip was again washed with a gentle stream of distilled water and then dipped in the double-distilled water.

### 4.7. Measurement of Secretory IgA in Saliva

**Figure 4:**
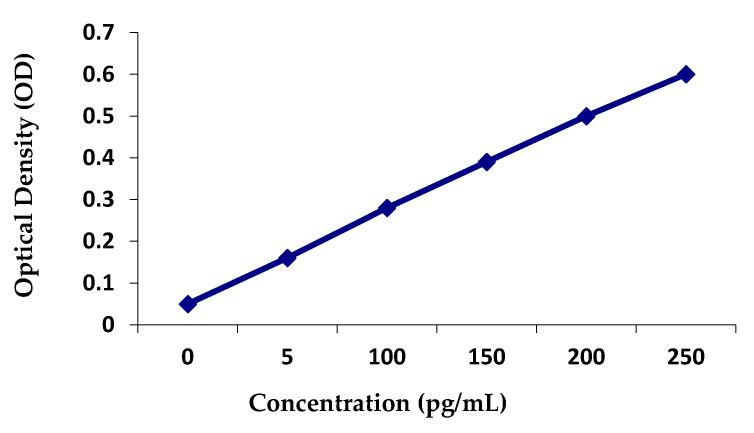
Standard curve of IgA.

Detection of IgA in saliva was performed by sandwich ELISA. In these assays, F96 microtitre plates were coated overnight at 4 °C with 0.2 µg/well of rabbit anti-IgA antibodies. Blocking was performed by the use of phosphate buffer containing 0.5% bovine serum albumin (BSA) at room temperature for 90 min. 100 µL saliva samples (in duplicate) and standard samples (in duplicate) were pipetted into the microtitre wells. The plates were incubated for 90 min at 37 °C. The wells were washed five times with a washing solution. Then, 100 µL of goat anti-human IgA conjugated with horseradish peroxidase (HRP) were pipetted into each well, and the plates were incubated for 30 min at 37 °C. The wells were washed five times with a washing solution and tapped dry. A fresh solution of substrate (tetramethylbenzidine, 100 µL) was added, the plates were incubated for 15 min at room temperature. The enzyme reaction was stopped. Salivary IgA levels were detected by use of a standard curve as shown in Figure 4.

## 5. Conclusions

According to the results obtained from the current study, the researchers noted that the thermoplastic retainers helped change the oral cavity environment by increasing IgA level and pH acidity of saliva while the microbiota status increased during the three months of thermoplastic retainer use studied. At the same time, there were increases in the number of *S. mutans* and *Lactobacillus* colonies. Finally, certain isolates were resistant toward CHX. The study will be continued by examining the changes in anaerobic bacteria and fungi during the three months of wearing a retainer.

## Figures and Tables

**Table 1:** The isolates percentages during three months.

First Month	Ratio	Second Month	Ratio	Third Month	Ratio
*Lactobacillus* spp.	100%	*Lactobacillus* spp.	100%	*Lactobacillus* spp.	100%
*Streptococcus* spp.	100%	*Streptococcus* spp.	100%	*Streptococcus* spp.	100%
*Staphylococcus aureus*	70%	*S. aureus*	80%	*S. hominis*	80%
*Neisseria* spp.	50%	*S. epidermidis*	50%	*S. saureus*	70%
*Staphylococcus epidermidis*	40%	*Neisseria* spp.	40%	*Neisseria* spp.	60%
*Micrococcus luteus*	30%	*M. luteus*	40%	*M. luteus*	60%
*Bacillus* spp.	10%	*Staphylococcus hominis*	20%	*Acinetobacter baumannii*	50%
		*Bacillus* spp.	20%	*S. epidermidis*	50%
				*Streptococcus thoraltensis*	40%
				*R. ornithinolytica*	40%
				*Bacillus* spp.	30%

**Table 2:** The secretory IgA concentration and pH salivary acidity for three months.

Months	IgA Levels (mg/dL)		Salivary pH	
	Control	Patients	Control	Patients
First month	8.2 ± 0.32 ^a^	8.4 ± 0.142 ^b^	6.6 ± 0.1 ^a^	6.5 ± 0.112 ^a^
Second month	8.1 ± 0.12 ^a^	13.4 ± 0.17 ^a^	6.7 ± 0.01 ^a^	6.2 ±0.312 ^a^
Third Month	7.9 ± 0.02 ^a^	13.8 ± 0.02 ^a^	6. 7 ± 0.21 ^a^	5.9± 0.26 ^a^

Different letters denote significant differences between the groups at *p* ≤ 0.05; Similar letters denoted no significant differences between the groups at *p* ≤ 0.05.

## Data Availability

Available within the manuscript in the form of tables and figures. Raw data can give upon reasonable request.
